# Metagenomic islands of hyperhalophiles: the case of *Salinibacter ruber*

**DOI:** 10.1186/1471-2164-10-570

**Published:** 2009-12-01

**Authors:** Lejla Pašić, Beltran Rodriguez-Mueller, Ana-Belen Martin-Cuadrado, Alex Mira, Forest Rohwer, Francisco Rodriguez-Valera

**Affiliations:** 1 Evolutionary Genomics Group, División de Microbiología, Universidad Miguel Hernández, Apartado 18, San Juan 03550, Alicante, Spain; 2 Department of Biology, San Diego State University, 5500 Campanile Drive, San Diego, CA 92182, USA; 3 Department of Biology, University of Ljubljana, Večna pot 111, 1000 Ljubljana, Slovenia

## Abstract

**Background:**

Saturated brines are extreme environments of low diversity. *Salinibacter ruber *is the only bacterium that inhabits this environment in significant numbers. In order to establish the extent of genetic diversity in natural populations of this microbe, the genomic sequence of reference strain DSM 13855 was compared to metagenomic fragments recovered from climax saltern crystallizers and obtained with 454 sequencing technology. This kind of analysis reveals the presence of metagenomic islands, i.e. highly variable regions among the different lineages in the population.

**Results:**

Three regions of the sequenced isolate were scarcely represented in the metagenome thus appearing to vary among co-occurring *S. ruber *cells. These metagenomic islands showed evidence of extensive genomic corruption with atypically low GC content, low coding density, high numbers of pseudogenes and short hypothetical proteins. A detailed analysis of island gene content showed that the genes in metagenomic island 1 code for cell surface polysaccharides. The strain-specific genes of metagenomic island 2 were found to be involved in biosynthesis of cell wall polysaccharide components. Finally, metagenomic island 3 was rich in DNA related enzymes.

**Conclusion:**

The genomic organisation of *S. ruber *variable genomic regions showed a number of convergences with genomic islands of marine microbes studied, being largely involved in variable cell surface traits. This variation at the level of cell envelopes in an environment devoid of grazing pressure probably reflects a global strategy of bacteria to escape phage predation.

## Background

Prokaryotic genomes are extraordinarily plastic entities and vary widely within the limits of a well defined species. In order to describe these large genetic reservoirs the pan-genome concept was introduced [1]. According to this concept, the species genome is composed of a core genome, containing genes present in all (or most) strains and a variable genome, containing genes present only in some strains.

In some cases, this variation is concentrated in hypervariable sets of genes, known as genomic islands [2-4]. Genomic island genes are often involved in specific lifestyles [5,6], e.g. symbiosis or pathogenesis [7,8] and frequently have the hallmarks of horizontally transferred genetic material such as different GC content or codon usage [9,10]. However, very little is known about the dynamic processes that originate and maintain the large genomic variability found in closely related prokaryotic genomes.

Metagenomics provides a new way to look at the dynamics and flexibility of prokaryotic genomes in nature [3,11-13]. When a microbe is well represented in an environment, and a metagenomic database from the same or a similar environment is available, it is possible to analyze genome recruitment - the preservation of genomic sequences in the natural environment. Using this approach, several authors working in different kinds of aquatic environments have found that some regions of sequenced genomes are poorly or not at all represented in the environment even when large stretches of the genome are nearly 100% similar to fragments from the metagenome [3,11-14]. In accordance with previous studies mentioned above, these genome stretches have been identified as genomic islands. However, this nomenclature is somewhat misleading. Although these islands often do correspond to classical genomic islands, identified through comparison of closely related prokaryotic genomes, there is not always a complete overlap [3,15]. Thus, although the latter often lack representation in the metagenome this is not always the case and vice-versa. In order to distinguish between these subtypes, we propose the term metagenomic island (MGI) to describe genome stretches identified by tiling of metagenomic reads against a reference strain genome.

To understand the mechanisms that generate the variability reflected by MGIs and their potential adaptive value [5,6], the gene content of metagenomic islands in different prokaryotic species needs to be explored. Microbial communities of extreme environments are especially appealing for this type of analysis. As a rule of thumb, these systems support low microbial diversity to the point of being dominated by few types of organisms with tightly defined population structure [16]. A typical example of extremely simplified microbial communities can be found in terminal pans of solar salterns where microorganisms endure saturated concentrations of NaCl. Known as crystallizers, these pans support very specialized hyperhalophilic archaea and bacteria [16,17]. The latter have been shown to be represented almost exclusively by *S. ruber *[17,18]. This hyperhalophilic member of CFB group is repeatedly reported in significant numbers from distinct hypersaline habitats around the world [18]. Comparative analysis of available 16S rRNA gene sequences indicated that *S. ruber *strains genetically differ and can be classified into at least two distinct phylotypes [18]. Here, we report for the first time the delimitation and a detailed description of *S. ruber *MGIs as seen by comparing type strain DSM 13855 genome with the metagenome of a solar saltern crystallizer. The results of this study display similarities with previously described metagenomic islands of another crystallizer species - the archaeon *Haloquadratum walsbyi *DSM 16790 as well as with MGIs of marine bacteria.

## Results

### Analysis of environmental genomic libraries

The metagenomic library used in this study was generated from environmental DNA obtained from crystallizer ponds of Chula Vista salterns, near San Diego, California on a GS20 sequencing platform. In total, 618127 reads were analyzed. The average read length was 100 bp. Using *E*>1e-5 BLASTX identity thresholds against the *nr *database we were able to phylogenetically assign approximately 10% of obtained reads. Several haloarchaeal species were found to be abundant and represented over 80% of assigned dataset. These were *H. walsbyi *(23% of reads), *Haloarcula marismortui *(23% of reads), *Natronomonas pharaonis *(20% of reads) and *Halorubrum lacusprofundi *(22% of reads). Bacteria were represented almost exclusively by *S. ruber *(12% of reads). The second set of metagenomic sequences (2974 sequences) was available from Legault et al. (2006) [11]. These authors used Sanger sequencing to end sequence a 2000 clone fosmid library constructed from samples of crystallizer brine of salterns in Santa Pola, Spain. The simple microbial community encountered in previous studies carried out here was composed of *H. walsbyi *(>80% of cells) and *S. ruber *(up to 20% of cells) [17].

Metagenomic reads of both datasets were tiled against available genomes in genome recruitment analysis using MUMmer. As expected, marine organisms and moderate halophiles did not recruit in metagenomes. In comparisons involving the Chula Vista salterns metagenome, significant recruitment was observed with genomic sequences of *S. ruber *and *H. walsbyi*. In consistence with results obtained by BLASTX analysis, over 10% of the dataset could be mapped to genome of *S. ruber *DSM 13855. The latter recruited a total of 90477 fragments (14.6% of entire dataset) out of which 17120 fragments were at 100% sequence identity. The genome of *H. walsbyi *DSM 16790 recruited a total of 56985 fragments out of which 11764 fragments gave hits at 100% sequence identity. This data confirmed the predominant role of these organisms in such hypersaline environments. The recruitment of remaining halophilic microorganism genomes in San Diego salterns was mostly moderate. Genomic sequence of *Halobacterium salinarum *R1, found scarce in BLASTX analysis, recruited 26135 reads with no recruitment observed above 97.5% sequence identity. Recruitment of presumably abundant species was only moderate. Genomic sequence of *H. marismortui *ATCC 43049 recruited 32416 fragments (1070 at 100% sequence identity), *H. lacusprofundi *ATCC 49239 recruited 41487 fragments (1646 at 100% sequence identity) and *N. pharaonis *DSM 2160 recruited 34933 fragments (1334 at 100% sequence identity). Together with BLASTX results these findings indicate that the sequenced members of the above genera are not well represented in this specific environment although some unknown relatives must be present. Not surprisingly, the above genomes originate from hypersaline environments other than salterns [19] namely the Dead Sea (*H. marismortui*), Antarctic Deep Lake (*H. lacusprofundi*), and highly saline soda lakes in Egypt and Kenya (*N. pharaonis*), while genomic sequences of highly recruiting *H. walsbyi *DSM 16790 and *S. ruber *DSM 13855 were determined from strains originally isolated from Spanish Mediterranean salterns [20-23].

Next, the same set of genomes was compared to Santa Pola dataset. No recruitment was observed with *S. ruber *DSM 13855 since the biomass collection procedure applied (filtration onto 2 μm pore size filters, see Methods) prevented collection of significant amounts of this microbe. In fact, genomic recruitment was observed only with *H. walsbyi *DSM 16790 as described and discussed before [11,12]. It is worth mentioning that the observed island pattern was very similar with both datasets (Additional file 1). These results indicate that metagenomic islands are a feature conserved within species regardless of geographic origin of the genomic sequence or metagenomic dataset. Furthermore, the phenomenon seems to be unaffected by the sequencing effort (within the ranges described here) or sequencing technique used.

### Genomic plasticity in *Salinibacter ruber *DSM 13855

**Figure 1:**
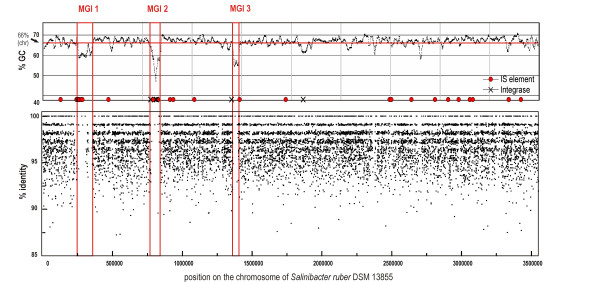
***Salinibacter ruber *DSM 13855 genome and metagenomic islands**. (a) GC-content of *Salinibacter ruber *genome plotted with a sliding window of 1000 nucleotides. Location of integrases and IS transposases along the genome are indicated. (b) Coverage of San Diego saltern crystallizer metagenomic reads. Individual metagenomic reads were aligned to the sequenced strain genome and the alignment-sequence conservation visualized in the form of percent identity plot. Each dot on the graph represents an individual sequence read aligned along its homologous region in *Salinibacter ruber *DSM 13855 genome. *Y *axis reflects its nucleotide percent identity to syntenic region. The regions lacking representation in the metagenome are boxed and described in the text as metagenomic islands.

When the sequencing reads were tiled against the genome of *S. ruber *DSM 13855 three MGIs where very few reads matched regions in the genome could be detected (Figure 1). When genomic recruitment analysis was examined using BLAST instead of MUMmer, the pattern observed was almost identical to that mentioned above (Additional file 2). This indicates that the observation is not biased by the methodology used.

**Figure 2:**
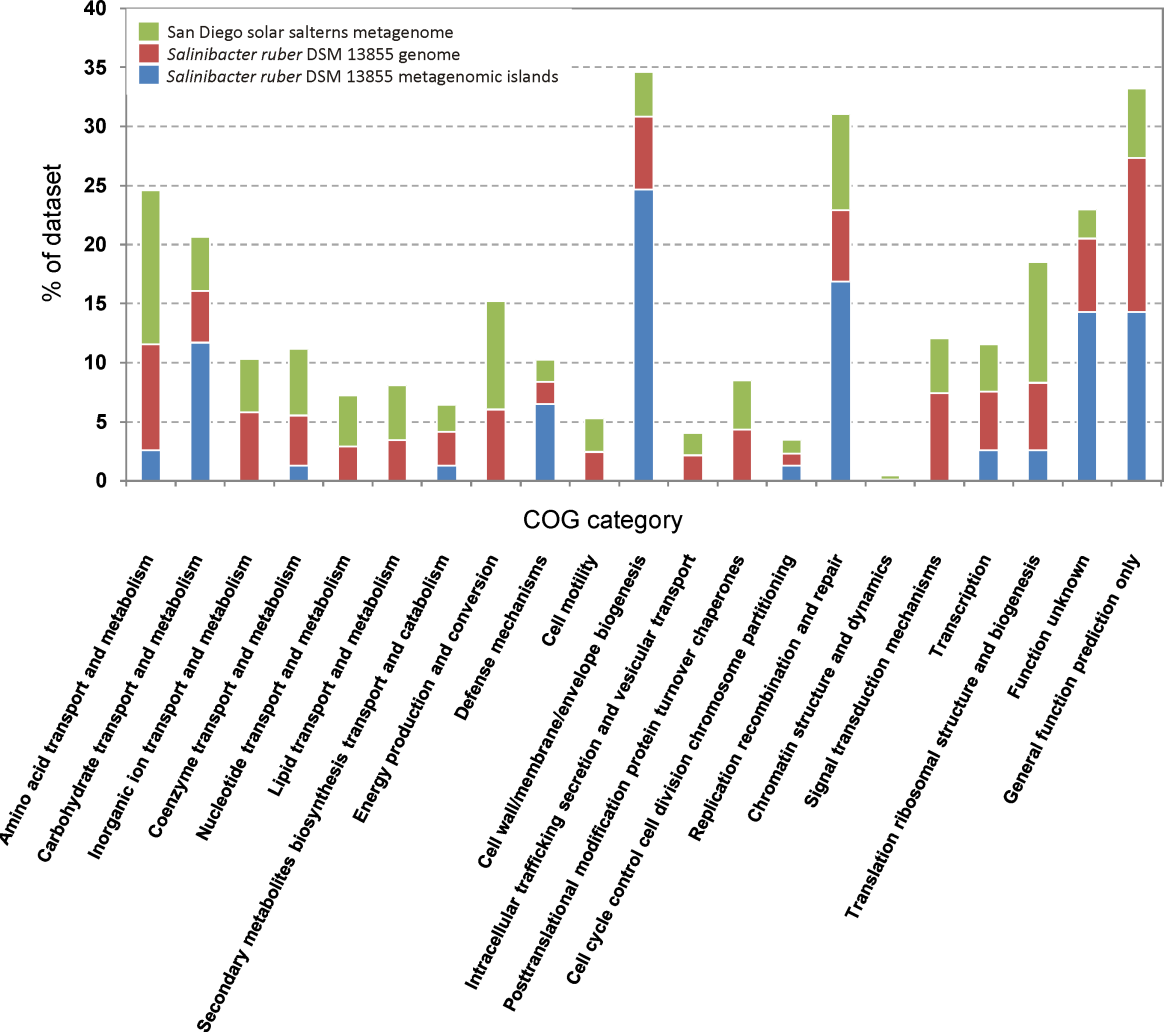
**Distribution of Clusters of Orthologous Groups (COGs) in *Salinibacter ruber *DSM 13855 metagenomic islands, genome of *Salinibacter ruber *DSM 13855 and San Diego crystallizer metagenome**.

Metagenomic islands showed several features typical of highly unstable genomic regions. They were characterized by atypical GC content (56% versus 66%), presence of pseudogenes and high numbers of short hypothetical proteins. The islands also contained three out of four phage integrases found in the *S. ruber *genome. Another notable feature of MGIs was a low average coding-region density of 54.0%, 64.7% and 45.4%, respectively, compared to 84.8% for the whole *S. ruber *genome. Furthermore, the majority of genes in the islands (54%, 66% and 88%, respectively) were most similar to species only distantly related to *S. ruber*. Finally, compared to the *S. ruber *genome and the San Diego metagenome, metagenomic islands were enriched in genes involved in carbohydrate transport and metabolism, cell wall/membrane/envelope biogenesis, recombination, replication and repair (Figure 2).

**Figure 3:**
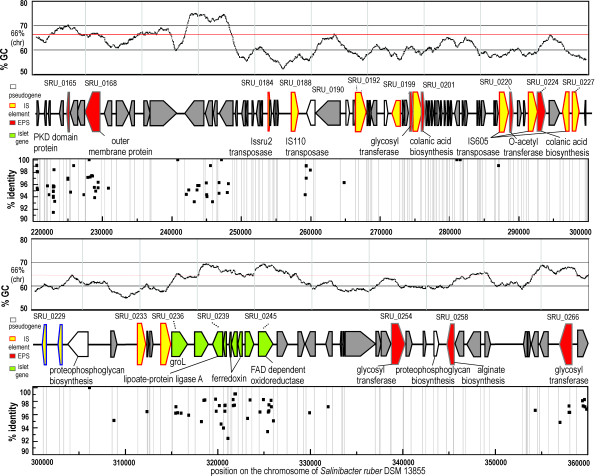
***Salinibacter ruber *DSM 13855 metagenomic island 1 and San Diego crystallizer metagenome**. GC-content of the island is plotted with a sliding window of 1000 nucleotides. Location of metagenomic island 1 on *S. ruber *DSM 13855 genome is indicated by nucleotide position number at the beginning and the end. ORF names are designated near each box.

MGI 1 (Figure 3) is 109 kbp long and is located between nucleotides 249037 and 358080 (ORF SRU_0178-SRU_0266) in the *S. ruber *genome. The observed correspondence between GC content and number of metagenomic hits is especially evident in MGI 1 in which a region of metagenomic hits is found corresponding with return to normal GC values. The MGI 1 genes appear to code for exopolysaccharide biosynthesis. These include two gene clusters annotated as colanic acid biosynthesis proteins (SRU_0201; SRU_0224), their respective glycosyl transferases (SRU_0199, SRU_0254 and SRU_0266) and acetyltransferase (SRU_0212). Colanic acid is a well known polysaccharide found in some *Escherichia coli *strains. There is evidence showing that this polysaccharide is recognized and degraded by some *E. coli *bacteriophages in their life-cycle [24]. Other genes found in MGI 1 include remnants of complete exopolysaccharide operons such as genes involved in biosynthesis of alginate (SRU_0258) and pseudogenes involved in biosynthesis of proteophosphoglycan (SRU_0231, SRU_0265).

**Figure 4:**
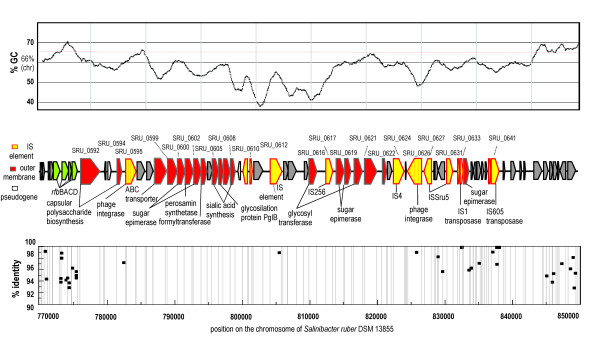
***Salinibacter ruber *DSM 13855 metagenomic island 2 and San Diego crystallizer metagenome**. GC-content of the island is plotted with a sliding window of 1000 nucleotides. Location of metagenomic island 2 on *S. ruber *DSM 13855 genome is indicated by nucleotide position number at the beginning and the end. ORF names are designated near each box.

MGI 2 (Figure 4) is 70 kbp long and is located between nucleotides 775936 and 845933 (ORF SRU_0592-SRU_0647) of the *S. ruber *genome. The genes of this island are organized in a tight cluster preceded by a phage integrase. Several genes of this region have been found in O-polysaccharide gene clusters of pathogenic Gram negative bacteria [25]. Such are two genes involved in synthesis of sialic acid (SRU_0605, SRU_0608), a sugar known to inhibit phage adsorption [26]; a perosamine synthetase gene (SRU_0601), a gene that is found in perosamine (4,6-dideoxy-D-mannose) containing repeat unit polysaccharides [25]; and a formyltransferase gene (SRU_0603), predicted to be involved in 4-formamido-4,6, dideoxymannose synthesis [25], another putative component of O-polysaccharide repeated units. This region also contains genes annotated as involved in colanic acid synthesis, five epimerases as well as glycosyl transferase genes. The latter are required for sequential transfer of nucleotide sugar precursors to form an oligosaccharide on a carrier lipid [25]. The region also contains genes involved in extracellular polysaccharide assembly, unit translocation across the membrane and subsequent polymerization. Two ORFs were identified as putatively involved in polysaccharide export. SRU_0592 is located at the very beginning of MGI 2 and contains three conserved *wza *domains, required for capsular polysaccharide translocation through the outer membrane in other Gram negatives [27], while SRU_0598 and SRU_0606 are ABC transporters. Furthermore, SRU_0594 shows features typical to that of a chain length determinant protein Wzz. It contains two transmembrane segments, located in the amino and carboxyl ends and a large periplasmic domain [28]. We were able to affiliate ORF SRU_0611 with O-chain polymerase on the bases of several notable features shared by this heterogeneous group of enzymes: domain similarity, high hydrophobicity of the gene product, protein topology (11 transmembrane segments) and presence of a characteristic cytoplasmic loop of approximately 30 amino acid residues [25]. However, we could not find the O-polysaccharide ligase WaaL that is in some species required for connecting the O-chain to the lipopolysaccharide core. In fact, the essential genes required to synthesize the core of the Gram negative lipopolysaccharide (i.e. COG0763, COG1043, COG1044, COG1663, COG0774) could not be found in this genome by comparison with other sequenced *Bacteroidetes *(where the genes coding for lipid A and structural polysaccharides are present) or by KEGG Pathway analysis, even under very permissive similarity thresholds. This indicates that the structure of the cell wall in *S. ruber *may be different from that in *Bacteroides *and *Porphyromonas*, that are close phylogenetic relatives. It is, therefore, very interesting that the organisation and composition of genes in MGI2 shares a number of similarities with O-polysaccharide gene clusters of pathogenic Gram negative bacteria [25]. The absence of core LPS genes would indicate that these external polysaccharides might be anchored by a non-canonical structure.

It is worth mentioning that the MGI 2 genes are preceded by *rfb*BACD - the genes involved in biosynthesis of dTDP-L-rhamnose, another component of O-polysaccharide repeat unit [25] and further upstream (cca. nucleotide 730000) by large clusters (*mur, fts*) involved in peptidoglycan synthesis. Due to the region hypervariability we hypothesize that the genes constituting MGI 2 are lineage dependent and perhaps unique to DSM 13855. In contrast to hypervariable MGI 2, the upstream peptidoglycan synthesis genes are well preserved in the metagenome and thus perhaps in all lineages of *S. ruber*. Although we could not find evidence of genes involved in the synthesis of the core lipopolysaccharide, the similarities shared between MGI 2 of *S. ruber *and O-polysaccharide gene clusters of other Gram negative bacteria indicate that MGI 2 genes could be involved in biosynthesis of extracellular polysaccharide component of the cell wall. We further hypothesize that this polysaccharide could be exposed on the outer surface of *S. ruber *cell wall.

**Figure 5:**
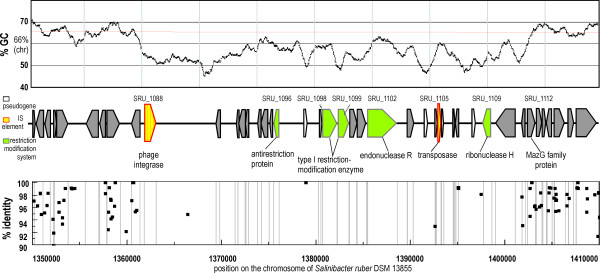
***Salinibacter ruber *DSM 13855 metagenomic island 3 and San Diego crystallizer metagenome**. GC-content of the island is plotted with a sliding window of 1000 nucleotides. Location of metagenomic island 3 on *S. ruber *DSM 13855 genome is indicated by nucleotide position number at the beginning and the end. ORF names are designated near each box.

MGI 3 (Figure 5) is located between nucleotides 1360489 and 1403241 (42.8 kbp) and includes ORFs SRU_1087 to SRU_1112. The island starts with a phage integrase and contains a mix of DNA related enzymes. The restriction-modification enzymes type I are represented by SRU_1098 (*Hin*dVIIp), SRU_1099 and SRU_1102 (Hsd family type). These genes are preceded by an ArdA antirestriction protein (SRU_1096). Studies suggest that ArdA proteins and type I restriction modification systems, may be involved in the control of gene transfer among bacterial genomes [29]. The island ends with a MazG protein, a nucleotide triphosphate pyrophosphohydrolase of unknown function which is highly conserved among bacteria [30]. Given the vast amount of presumably noncoding DNA within the MGI 3, we have searched for pseudogenes - sequences that showed similarity to a sequence classified as a gene in another species (*E *< 1e-20) but in which frameshift and substitution mutations to stop codons have started to accumulate [31]. However, only four genes were identified using this criterion, three were classified as transposases and one as the catalytic subunit of phage integrases. Given the available data, we conclude that this island might contain remnants of a lysogenic phage inserted in *S. ruber *genome and absent in most cells in the natural environment.

### Convergence with MGIs of other microorganisms

Comparative analysis of hypervariable regions detected in this analysis was performed using genomes available from GenBank ftp://ftp.ncbi.nih.gov/genomes/ and metagenomic datasets available from this study and Camera database http://camera.calit2.net/index.php. In this analysis the metagenomic islands of *S. ruber *DSM 13855 showed several convergences with metagenomic islands of other microbes studied, in particular high numbers of hypothetical and conserved hypothetical proteins, transposases, integrases and transport-related proteins.

A metagenomic island enriched in products involved in restriction/modification and DNA repair was a feature shared by MGIs of *S. ruber*, *H. walsbyi *[12], *Prochlorococcus marinus *[3], *Candidatus *Accumulibacter phosphatis [32] and *Ferroplasma acidarmanus *[33]. These MGIs are often associated with phage-type integrase genes and might have developed as a result of prophage insertion.

The presence of metagenomic islands putatively involved in biosynthesis of polysaccharide component of cell wall was a feature shared by MGIs of *S. ruber *and most Gram negative aquatic microbes such as *P. marinus *[3], *Candidatus *Pelagibacter ubique [4], *Synechococcus *sp. WH8102 and *Synechococcus *sp.CC9311 [34]. In addition, presence of variable genes involved in extracellular polysaccharide biosynthesis was reported from *Candidatus *Accumulibacter phosphatis [32] and *Ferroplasma acidarmanus *[33]. Interestingly, recruitment studies of *H. walsbyi *[12,14], an archaeon with glycoprotein S-layer based cell wall, showed the presence of at least two MGIs putatively involved in the synthesis of the cell wall.

## Discussion

One of the most effective ways to study genomic plasticity in prokaryotes is to compare metagenomic data to the genomes of strains present in the environment studied [3,11-13,32-34]. In this study, this approach was applied to an extreme hypersaline environment, the brine of a solar saltern. Good recruitment properties were only observed when genomic sequences of strains isolated from a similar environment were compared to the metagenome. In this particular case the strains recruiting efficiently were isolated from other geographically solar salterns. In all cases, representative genomes possessed a typical recruiting pattern with metagenomic islands as their most remarkable feature.

It seems to be a general phenomenon of many, if not most, bacteria that a large part of the gene cluster coding for the polysaccharide component of cell wall is extremely variable. In clinical isolates, this phenomenon has been known for many years, more than 180 lipopolysaccharide serotypes have been described in *Escherichia coli *and more than 50 in *Salmonella enterica *[25]. As mentioned above, the presence of genes involved in the synthesis of the polysaccharide component of cell wall was a feature shared by variable regions of *S. ruber*, *P. marinus*, *Candidatus* Pelagibacter ubique and *Candidatus *Accumulibacter phosphatis. In Candidatus *Accumulibacter phosphatis *sludge bioreactors the variation in dominant lineages was noted not only in the exopolysaccharide synthesis cluster genes but also in clustered regularly interspaced short palindromic repeat (CRISPR) elements [35]. These elements, regularly interspaced by foreign DNA sequences, can provide immunity to the phages from which they were derived [36]. However, this strategy appears less widespread in brines since we were not able to identify any CRISPR in genome of *S. ruber *while *H. walsbyi *genome contained only one such element. Likewise, these elements were scarce in the metagenomes studied.

The extreme environment of solar saltern crystallizer supports dense yet simple microbial communities composed of highly related strains of dominant species [16]. Such environments do not host phagotrophic protists, remain free from grazing pressure and are natural targets for phage predation [37,38]. We hypothesise that cell wall polysaccharide variability supplied by metagenomic islands could play a role in defence against this predation. In the past, phages have been shown to target lipopolysaccharide through their host recognition machineries [39] or strain-specific polysaccharases [24]. In the specific case of *S. ruber*, several components of MGI 1 and particularly MGI 2 indicate this type of strategy. They include genes involved in biosynthesis of colanic acid, shown to be hydrolysed by phage induced enzymes in *Escherichia coli *[24], and sialic acid biosynthesis genes, reported to be a part of phage receptors [39]. In densely populated aquatic habitats such genes will be subject to arm races (also known as Red Queen strategies), and be required to be as plastic as their bacteriophage counterparts to maintain a reasonable population density and avoid catastrophic crashes of the population due to phage lysis. This hypothesis is supported by results showing high expression of metagenomic island genes suggesting that they encode proteins central to cellular processes in specific genotypes [13]. In order to achieve the desired level of genome plasticity as least two mechanisms could be employed. Metagenomic islands are transposase rich areas in which genes often share homology with multiple phylogenetically diverse microbes and thus might act as lateral gene transfer hot spots in order to achieve the observed level of genome plasticity. Additional diversification through lateral gene transfer and recombination could be achieved through modular organisation of cell wall polysaccharide biosynthesis genes. This was observed in genome of *S. ruber *where a lineage-specific set of genes, located within the metagenomic island, is preceded by *rfb *gene cluster involved in rhamnose biosynthesis and further upstream by *mur *and *fts *clusters involved in peptidoglycan synthesis. This phenomenon has been noted in at least one another species. In *Streptococcus thermophilus*, a Gram positive species and therefore devoid of lipopolysaccharide, the exocellular polysaccharide biosynthesis cluster is composed of core gene cluster, represented by *deo*D-*eps*ABCD, and followed by a variable region [40]. Interestingly, similar to crystallizer brine, the natural environment of *Streptococcus thermophilus *also supports dense microbial communities with low microbial diversity that is devoid of protists grazing.

## Conclusion

Tiling the genomic sequence of *S. ruber *DSM 13855 against reads from the San Diego saltern crystallizer metagenome has shown that the conserved backbone of this genome is well represented in the metagenomic data. This result is quite remarkable because this isolate comes from a Mediterranean solar saltern. However, like other microbial genomes when compared to a metagenome in which they are well-represented the tiling of the genome leaves empty regions of low coverage or metagenomic islands.

Metagenomic islands share several features with classical genomic islands described by comparing genomes of closely related strains such as atypical GC content, high frequency of phage/IS elements and hypothetical genes. However, their gene content appears largely involved in biosynthesis of cell wall polysaccharides. This phenomenon appears to be general in this and other marine microbes studied and might reflect a global strategy of bacteria to escape phage predation [14].

## Methods

### Genomic libraries and sequencing

The environmental genomic sequences collected from Santa Pola solar salterns (Alicante, Spain) were obtained in a previous study as described in [11]. The DNA was extracted from biomass retained on a 2 μm pore size filter. A 2000 clone fosmid library was end sequenced resulting in 2947 available sequences.

The environmental genomic sequences collected from Chula Vista solar salterns (Chula Vista, CA), were obtained from biomass retained on a 0.2 μm pore size tangential flow filter and were sequenced by pyrosequencing on a GS20 sequencing platform (454 Life Sciences, CT, USA). A total of 618127 reads of average length of 100 bp were obtained.

### Sequence analysis

#### Raw sequence screening and analysis

The raw metagenomic sequence obtained from Chula Vista solar salterns was screened to remove low quality and short sequences. To this aim the software The Hairdresser was developed (see Availability and requirements section below). To this aim the software The Hairdresser was developed (see Availability and requirements section below). Using the multifasta metagenomic sequence file as input variable, the software enables removal of sequences of desired length from metagenomic sequence file using the ShortCut function, removal of desired subset of the metagenomic sequence file using the ClipOut function, renames sequences using the ReStyle function and calculates thermostability index of the metagenomic sequence file entries using the HotComb function.

#### Recruitment analysis

A total of 2947 sequences available from Santa Pola solar salterns and 618127 reads available from San Diego solar salterns were aligned against reference genomes by using the MUMmer program version 3.19 [41]. Specifically, to calculate alignments 'PROmer' program with the 'maxmatch' option was used. The percent identity plots were generated using 'mummerplot'.

For BLAST-based recruitment analysis, the genome was split into fragments of 50 nucleotides in length and compared to the metagenome using basic local alignment search tool BLASTN (DNA vs. protein) [42]. The plot was generated by counting the number of hits to each fragment versus position on the chromosome.

#### Annotation of islands

Island genes were re-annotated to ensure no open reading frame (ORF) was missed. Protein coded genes were predicted using the annotation package GLIMMER [43], and were further manually curated. Spacers were subsequently searched against the non-redundant database using BLAST [42]. ORFs were compared to known proteins in the non-redundant database using the BLASTX program (translated DNA vs. protein). All hits with *E*-value greater than 10^-5 ^were considered non-significant.

#### Sequence analysis

Additional BLASTN, BLASTP and PSI-BLAST searches were performed when needed. All hits with *E*-value greater than 10^-5 ^were considered non-significant. COG classification of *S. ruber *DSM 13855 genomic sequences was obtained from GenBank. COG classification of metagenomic sequence reads was performed by conducting rps-blast search against the COG database. Significant sequences were distributed in COG categories. KEGG pathway analysis was available from http://www.genome.jp/kegg/pathway.html. GC content was identified using the 'geecee' program from EMBOSS package [44]. GC plots were generated using 'insilico' web server http://insilico.ehu.es. Protein topology predictions were performed using SOSUI, PredictProtein and HMMTOP available from Expasy proteomics server http://www.expasy.ch/. Conserved blocks in groups of unaligned protein sequences were identified by using the Block Maker program http://blocks.fhcrc.org/blockmkr/make_blocks.html. CRISPR analysis was performed using CRISPR finder available from http://crispr.u-psud.fr/crispr/CRISPRdatabase.php?page=own. Genes were identified as pseudogenes when they showed similarity to a sequence classified as a gene in another species (*E *< 1e-20) but in which frameshift and substitution mutations to stop codons have started to accumulate [30].

#### Accession numbers

The sequence of the complete genome of *Haloquadratum walsbyi *DSM 16790 was deposited as [GenBank:AM180088.1, GenBank:AM180089.1], the sequence of the complete genome of *Salinibacter ruber *DSM 13855 was deposited as [GenBank:NC_007677, GenBank:NC_007678], the metagenomic sequences of Santa Pola salterns were deposited as [GenBank:DU826964-DU824018] and the metagenomic sequences of San Diego solar salterns were available through http://scums.sdsu.edu/.

## Availability and requirements

*The Hairdresser *software requires the Microsoft Windows Vista or XP operating systems. The program was written with Borland Delphi 7 Enterprise and the executable file, source code and example files are available as Additional File 3 and at the following open-source repository: http://hairdresser.sourceforge.net/.

## Authors' contributions

FR-V, FR and BRB conceived the work. BRB and FR contributed metagenomic sequence data from Chula Vista solar salterns. LP performed raw sequence data screening and analysis and performed phylogenetical analysis. ABMC carried out annotation of island genes. LP, BRB, AM and ABMC performed MUMmer analysis and contributed further bioinformatical analysis. LP made the figures. LP, ABMC and FR-V wrote the manuscript. All the authors read and approved the final manuscript.

## Supplementary Material

Additional file 1 ***Haloquadratum walsbyi *DSM 16790 genome and metagenomic islands. (a) GC-content of *Haloquadratum walsbyi *genome plotted with a sliding window of 1000 nucleotides**. Location of integrases and IS transposases along the genome are indicated. (b) Coverage of Santa Pola saltern crystallizer metagenomic reads. (c) Coverage of San Diego saltern crystallizer metagenomic reads Individual metagenomic reads were aligned to the sequenced strain genome and the alignment-sequence conservation visualized in the form of percent identity plot. Each dot on the graph represents an individual sequence read aligned along its homologous region in *Haloquadratum walsbyi *DSM 16790 genome. *Y *axis reflects its nucleotide percent identity to syntenic region. The regions lacking representation in the metagenome are boxed and described in the text as metagenomic islands. Click here for file

Additional file 2 ***Salinibacter ruber *DSM 13855 genome and metagenomic islands**. (a) Coverage of San Diego saltern crystallizer metagenomic reads as revealed by MUMmer analysis. *Y *axis reflects its nucleotide percent identity to syntenic region. (b) Coverage of Santa Pola saltern crystallizer metagenomic reads as revealed by BLAST analysis. *Y *axis reflects number of hits to syntenic region. The regions lacking representation in the metagenome are boxed and described in the text as metagenomic islands. Click here for file

Additional file 3 ***The Hairdresser *software**. Executable file, source code and example files of *The Hairdresser *software. Click here for file
