# Supplementary figures and images for: Metagenomic islands of hyperhalophiles: the case of Salinibacter ruber
Source: BMC Genomics. 2009 Dec 1;10:570. doi: 10.1186/1471-2164-10-570 (PMC2800850; doi:10.1186/1471-2164-10-570)

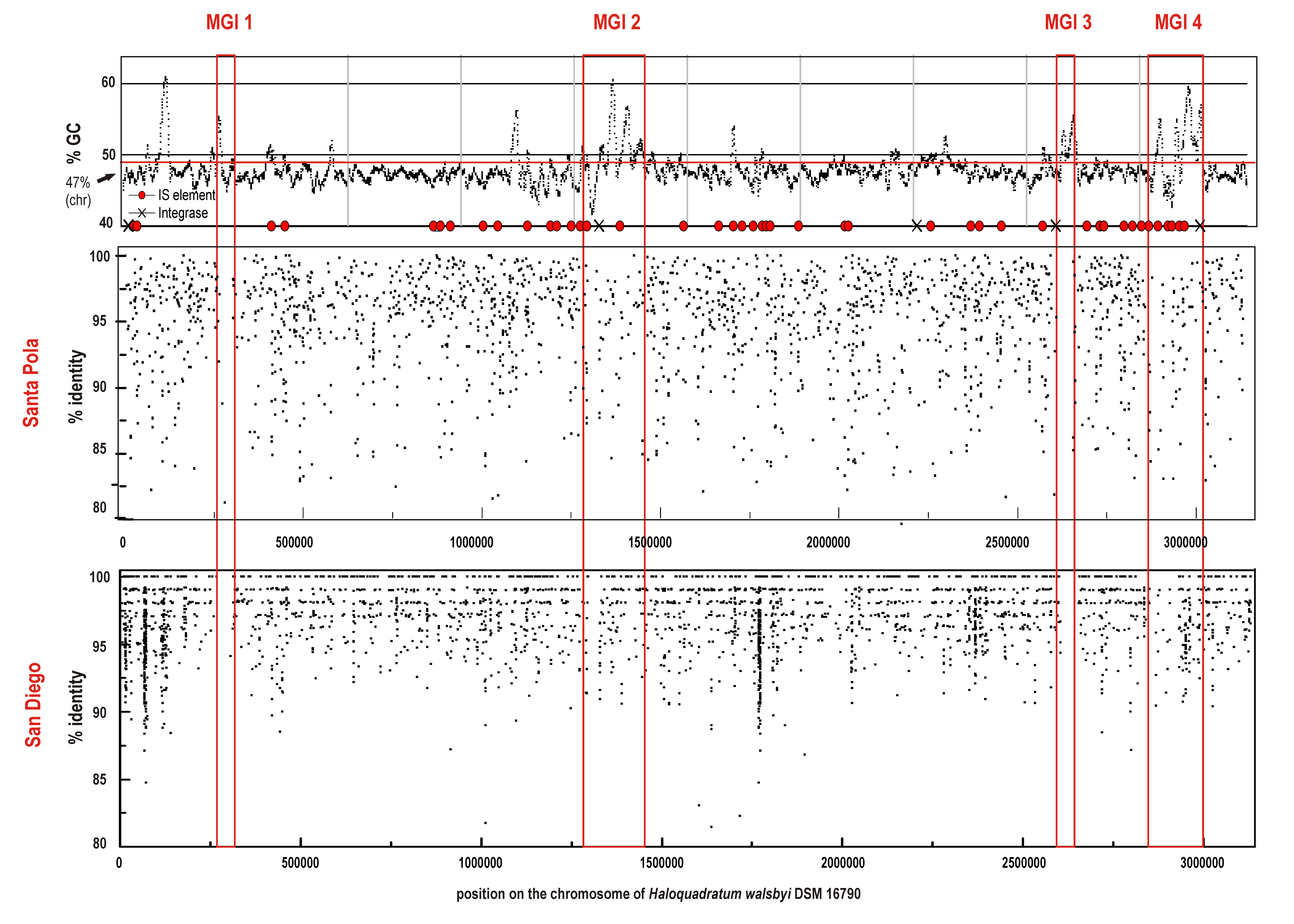

Supplement: Additional file 1 — Haloquadratum walsbyi DSM 16790 genome and metagenomic islands. (a) GC-content of Haloquadratum walsbyi genome plotted with a sliding window of 1000 nucleotides. Location of integrases and IS transposases along the genome are indicated. (b) Coverage of Santa Pola saltern crystallizer metagenomic reads. (c) Coverage of San Diego saltern crystallizer metagenomic reads Individual metagenomic reads were aligned to the sequenced strain genome and the alignment-sequence conservation visualized in the form of percent identity plot. Each dot on the graph represents an individual sequence read aligned along its homologous region in Haloquadratum walsbyi DSM 16790 genome. Y axis reflects its nucleotide percent identity to syntenic region. The regions lacking representation in the metagenome are boxed and described in the text as metagenomic islands. [file 1471-2164-10-570-S1.TIFF]

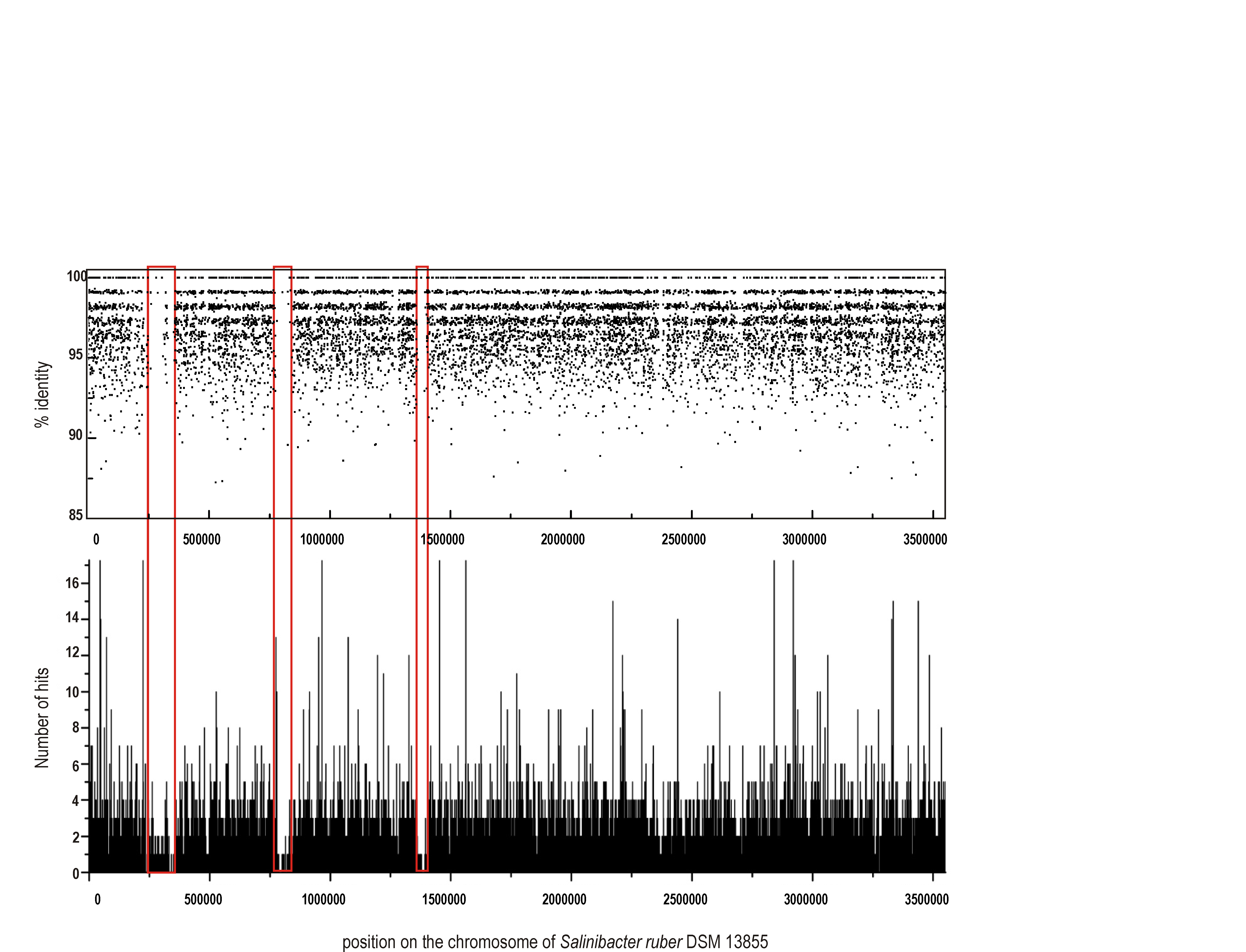

Supplement: Additional file 2 — Salinibacter ruber DSM 13855 genome and metagenomic islands. (a) Coverage of San Diego saltern crystallizer metagenomic reads as revealed by MUMmer analysis. Y axis reflects its nucleotide percent identity to syntenic region. (b) Coverage of Santa Pola saltern crystallizer metagenomic reads as revealed by BLAST analysis. Y axis reflects number of hits to syntenic region. The regions lacking representation in the metagenome are boxed and described in the text as metagenomic islands. [file 1471-2164-10-570-S2.TIFF]
